# O01 Post-transplant lymphoproliferative disorder (PTLD) in a patient with rheumatoid arthritis

**DOI:** 10.1093/rap/rkab067

**Published:** 2021-10-19

**Authors:** Sung-Hee Kim, Christopher Mortlock, Edmond Antaki, Alexander Zervudachi, Matilda Llewellin-Robinson, Jessica Gunn

**Affiliations:** Gloucestershire NHS Trust, Cheltenham, United Kingdom

## Abstract

**Case report - Introduction:**

Post-transplant lymphoproliferative disorder (PTLD) is defined as abnormal lymphoid proliferation related to immunosuppression, almost always associated with EBV. It ranges from benign mononucleosis to non-Hodgkin’s Lymphoma but is often used to describe the neoplastic syndromes. The exact pathophysiology is unknown but it is thought to be secondary to immunosuppression leading to lack of critical T cell control of B cell growth and thus unchecked proliferation of EBV-infected B cells resulting in malignancy. PTLD is a well-recognised phenomenon in transplant medicine but not many cases have been reported within the rheumatology patient population despite many on long term immunomodulatory therapy.

**Case report - Case description:**

A 53-year-old woman with a 15-year history of rheumatoid factor positive, erosive inflammatory arthritis and autoimmune hepatitis, on long term azathioprine and low dose prednisolone, presented with increasing arthralgia, intermittent skin rashes “like spots” and recurrent “throat infections”. She had active inflammatory arthritis, CRP of 63 and DAS28 score of 5.58. A decision was made to escalate to biologic therapy.

A chest radiograph demonstrated left lower zone opacity and subsequent CT TAP reported saddle PE, multiple bilateral lung nodules with cavitation, liver and renal lesions with extensive pericolic inflammation. She was commenced on anticoagulation and, whilst awaiting further tests, developed a fever with raised acute phase reactants. *Listeria monocytogenes* was ultimately isolated from blood cultures.

Two weeks later, despite commencing appropriate intravenous antibiotics, she experienced a partial seizure. An MRI of her brain revealed a rim-enhancing lesion in the right parietal lobe with extensive oedema and mass effect. There was also persistence of the multi-organ abnormalities on cross sectional imaging including PET CT. Extensive additional immunologic and microbiological testing revealed an atypical ANCA positivity (MPO and PR3 negative) with a low titre rheumatoid factor (16), negative anti CCP, positive ANA but negative ENA and dsDNA with normal complements. Her EBV IgG was positive, IgM negative but she had an EBV viral load of 714 copies/mL.

Numerous attempts were made to obtain definitive histology: an initial CT-guided lung biopsy reported non-diagnostic necrotic tissue; a subsequent VATS-guided biopsy of a lung lesion revealed histology suggestive of a rheumatoid nodule. A brain biopsy was deemed too high risk.  A liver biopsy ultimately demonstrated features on a spectrum between polymorphic post-transplant lymphoproliferative disorder (P-PTLD) and a monomorphic B-cell PTLD resembling EBV+ diffuse large B-cell lymphoma.

She was commenced on high dose methotrexate and R-CHOP chemotherapy as per haematology/oncology MDT.

**Case report - Discussion:**

This case posed a number of clinical conundrums.  Firstly, a wide differential diagnosis including infection (Listeria cerebritis), extra-articular manifestations of her rheumatoid arthritis (RA), including rheumatoid vasculitis, metastatic malignancy or a lymphoproliferative disorder. It was therefore crucial to continually review and reconsider the diagnosis in light of new information.

The second issue is consideration of opportunistic infection in a patient on long-term immunosuppression. We rarely encounter listeriosis and are consequently unfamiliar with the way the disease manifests itself in the context of immunosuppressed patients. This case posed a question of how we can monitor for over-immune suppression; whether leukocyte count is a reliable measure of the patient’s immune system or whether the frequency of infection is more a sensitive marker.

Thirdly, the role of EBV monitoring in this case is also interesting. We do not routinely measure EBV titres at time of initiation of DMARD therapy and we do not measure viral load. However, EBV DNA measurement has now been incorporated into routine practice in transplant medicine to help diagnose and monitor for PTLD in immunocompromised recipients. Patients identified as high risk undergo reduction in immunosuppression to prevent progression into PTLD. Unfortunately, a meta-analysis by AlDabbagh et al showed that there is no role in antiviral prophylaxis to prevent progression to PTLD.

Finally, advances in therapeutics have resulted in a large cohort of ‘stable’ patients with chronic autoimmune disease who require follow up in secondary care. A mismatch in resource and staff allocation means that often these patients can wait longer than intended for their planned follow up appointments. However, as demonstrated in this case, this patient group carries a risk of long-term immunosuppression, opportunistic infection and lymphoproliferative disorders and it is crucial to ensure that this group of patients is not overlooked.

**Case report - Key learning points:**

Lymphoma incidence in RA is increased by approximately two-fold compared to baseline population. The mechanism of causality is poorly understood but is postulated to be due to chronic stimulation of B cells by antigen or a virus, local inflammation and immune suppression leading to decreased surveillance by T cells. Currently, it is unclear whether the increased risk is due to immunosuppression or the severity of disease given that severe diseases are usually treated with more aggressive immunosuppression.  This poses a dilemma when treating RA-associated PTLD: to withdraw immunosuppressive therapy or treat RA more aggressively.

Kaiser et al.’s systematic review of lymphoma incidence in RA patients demonstrated 20 out of 26 studies reporting an increased incidence of lymphoma in RA and eight studies reporting a positive correlation between DMARD use and increased incidence of lymphoma. Furthermore, these studies, although underpowered, suggest that azathioprine and TNF inhibitors were more likely to be associated with lymphomas. Only 3 studies investigated the role of EBV, which was inconclusive.

Similarly, there is a paucity of literature on the role of EBV and lymphoproliferative disorders in rheumatology patients on long-term immunomodulatory therapy and further research is needed so that we can mitigate this risk for our patients.

In summary, PTLD is a rare, sometimes rapidly progressive and fatal, complication of RA in patients on long-term immunomodulatory therapy. Consequently, it is important to be alert to, and consider, this as a differential diagnosis and obtain appropriate tissue samples for histological confirmation.

Furthermore, this case also highlights the real risk of opportunistic infection amongst our patient population in addition to the need to carefully monitor this so called ‘stable’ cohort of patients to ensure early detection of potential complications of both long-term autoimmune disease and immunomodulatory therapy with de-escalation of the latter where appropriate.

